# Moving from Needs Assessment to Intervention: Fathers’ Perspectives on Their Needs and Support for Talk with Teens about Sex

**DOI:** 10.3390/ijerph19063315

**Published:** 2022-03-11

**Authors:** Jennifer M. Grossman, Amanda M. Richer, Belinda F. Hernandez, Christine M. Markham

**Affiliations:** 1 Wellesley Centers for Women, Wellesley College, Wellesley, MA 02481, USA; aricher@wellesley.edu; 2 University of Texas Health Science Center at Houston School of Public Health, Houston, TX 77030, USA; belinda.hernandez@uth.tmc.edu (B.F.H.); christine.markham@uth.tmc.edu (C.M.M.)

**Keywords:** reproductive health, adolescent health, father–child relations, needs assessment, psychosocial intervention

## Abstract

Talk with fathers about sex and relationships can support teens’ health, but its impact is limited as few fathers talk with their teens about sexual issues. Needs assessment and fathers’ input on intervention content and structure can guide the development of programs that support fathers’ health-promoting talk with their teen children about sex and relationships. In the present study, we explored fathers’ goals in their talk with teens about sex and relationships and barriers they perceive to these conversations, as well as what they would look for in an intervention program. Content analysis was conducted using interviews in the U.S. with 43 fathers of high school-aged teens (age 14–18). Themes explored fathers’ roles in talk with teens, key messages to teens, and approaches and barriers to conversations, in addition to attitudes toward an intervention, and feedback on intervention structure, content, and process. The findings suggest that fathers see talk with teens about sex as part of their roles, but face challenges in accomplishing this goal. Fathers’ feedback highlights their openness to an intervention and can guide the development of a peer-based and interactive program that addresses how to talk with teens about sex in addition to the content of these conversations.

## 1. Introduction

Adolescents’ risky sexual behaviors, such as sex without a condom and having multiple partners, have high costs, including transmission of sexually transmitted infections (STIs) and unintended pregnancy [1]. Young people aged 15–24 years acquire half of all new STIs in the U.S., and rates of gonorrhea and chlamydia are increasing for adolescents ages 15–19 [2]. Fathers’ talk with teens about sex can protect teens from risky sexual behavior [3,4]. However, the positive impact is limited as few fathers talk with their teens about sex, and those who do talk report low frequency [4]. Most research on parents’ communication with teens about sex focuses on mothers or includes communication with an unspecified “parent” [4,5]. While interventions to promote parent–teen talk about sex can reduce adolescent sexual risk behavior [6,7], few programs are tailored to support fathers’ health-promoting talk with teens about sex and relationships. To develop interventions that effectively support fathers’ talk with their teen children about sex and relationships, a better understanding of fathers’ goals for and perceptions of talk with their teens about sexual issues and what interferes with these conversations is needed. With few exceptions [8], little research explores barriers to fathers’ talk with teens about sex and relationships and their preferences for support for these conversations. To address these gaps, the current paper provides a qualitative exploration of fathers’ perspectives on their role in talking with their teens about sex, the barriers to these conversations, and what they would like to see in an intervention to support father–teen talk about sex and relationships.

Fathers’ engagement and support can positively influence teens and early adults’ sexual health over and above the influence of mothers [3,9,10]. Findings on the effects of fathers’ talk with teens about sex have been mixed. Fathers’ talk with teens about sex can protect teens from risky sexual behavior [3,4], although associations between communication and sexual behavior may be stronger for mothers than fathers [5]. One review of research found findings for positive associations between father–teen talk about sex and increased condom use and delayed sex [3], while another identified inconsistent findings for associations between father–teen talk about sex and teens’ sexual behavior [4].

The protective potential of father–teen talk about sex is reduced by fathers’ lack of talk with teens about sexual issues. Most teens report that fathers talk rarely or not at all with them about sexual issues [4]. When fathers talk with teens, conversations often address delaying sex, and include information on dating and relationships, and warning about HIV/AIDS and STIs [11]. These conversations also include how to use a condom and prevent pregnancy [12]. Fathers talk more about sexual topics with sons than with daughters [4,13], perhaps due to discomfort talking with daughters about sexual issues. or fathers’ beliefs that they have more responsibility for the sexual socialization of sons than daughters [14]. One study found that over half of daughters reported no communication from their fathers about risks of pregnancy, how to prevent HIV and STIs, and how to use condoms [15].

Low rates of fathers’ talk with teens about sex may be partially due to fathers’ perceived barriers to these conversations. Barriers to fathers’ talk with their teens about sex include discomfort talking about sexual topics, lack of clarity on how to initiate conversations, and uncertainty about their children’s developmental readiness for these discussions [16,17,18,19]. Fathers’ discomfort talking about sex may prevent them from initiating these conversations, as fathers who are comfortable talking with their teens about sex are also more likely to talk with their teens about sex [4]. Fathers also express concerns that their children may see them as intrusive if they initiate conversations and do not want to invade their privacy, and therefore may avoid starting conversations [16]. A study of female adolescents found that they perceived their fathers’ discomfort talking about sex and their avoidance of talking about sexual issues as interfering with their fathers’ effective communication about sex [20]. In addition, one study found that fathers are less likely to talk with their teens about sex when the teen’s mother shows high levels of maternal control [21], suggesting that the family system may also contribute to low levels of father–teen talk about sex.

Findings for parent-based sexual health interventions are mixed [22,23], but can increase parent–teen communication and comfort talking about sex [23,24,25] and reduce teen sexual risk behavior, including increased condom use and delayed sex [26,27]. However, few interventions uniquely support fathers’ preparedness for and engagement with health-promoting talk with teens about sex and relationships. Different approaches may be needed compared to programs with mothers to maximize fathers’ engagement with father–teen communication about sex. For example, fathers want guidance on specific sexual and reproductive health information and how to communicate about it with their teens [16]. Community-based approaches, such as developing a barber-led sex education program to support Black male teens and their fathers, may bring new opportunities for intervention [28]. Fathers may also benefit from a focus on indirect communication, such as alluding to sexual behavior without using clear sexual language or referring to a TV show or movie as a way to bring up a sexual issue [29,30]. A study found that more than half of fathers talked in indirect ways with their teens about sex and this indirect talk was associated with teens’ sexual behavior [30]. Fathers’ perspectives on what roles they want to play in talk with teens about sex and what interferes with these conversations can help shape father-focused interventions to support teens’ health. This fits with findings that the development of intervention programs benefits from community feedback on program content and structure and can increase the likelihood that the target group will participate in the program and that it will effectively meet their health needs [31].

Research showing that fathers’ talk with teens about sex and relationships can be protective, yet is limited by infrequent talk about sexual topics, suggests the need to develop interventions to support this communication. To work towards this goal, a better understanding is needed of fathers’ health-related goals for talk with teens about sex and relationships and the barriers to reaching these goals.

The PRECEDE/PROCEED model identifies steps toward the development of a health intervention program, which entails key collaboration with the community who experiences a health problem [32]. This paper focuses on steps of the PRECEDE component, which involve a needs assessment before an intervention is developed. It entails obtaining community input on health-related goals and priorities as well as barriers to achieving them [32]. To guide next steps in intervention development, this paper will also include fathers’ feedback on what they would look for in an intervention to support their talk with their teens about sex and relationships and their attitudes toward a potential intervention.

The objectives of the paper are to conduct a needs assessment to explore and understand fathers’ perspectives on their experiences promoting communication with their children on issues related to sex and to explore what fathers would look for in an intervention to promote this communication. Our guiding research questions are as follows. Research question 1: How do fathers view their goals, priorities, and barriers to talk with their teens about sex and relationships? Research question 2: What would fathers look for in an intervention to support their health-promoting talk with their teens about sex and relationships?

## 2. Materials and Methods

### 2.1. Study Design

In the current study, we used an exploratory, qualitative design as described by Stebbins [33]. Content analysis was used for data analysis in order to identify and interpret patterns in the data to explain a phenomenon [34]. This approach was used to explore fathers’ views on talk with their teens about sex and what fathers would like to see in an intervention to support father–teen communication. Consistent with conventional content analysis and limited prior research conducting needs assessments for fathers’ talk with their teens about sex and relationships, we did not use preconceived theoretical categories for data coding [35].

### 2.2. Participants and Environment

We collaborated with multiple community partners across the U.S. to recruit fathers for this interview study. We distributed flyers online (in both English and Spanish) and participated in Zoom meetings and presentations with our partners to help recruit fathers to this study. Fathers were instructed to complete an online survey to determine their eligibility for the study and provide contact information for the interview. Inclusion criteria were identifying as a father (biological, step, adoptive, or foster) of a high school-aged teen and having a relationship with the teen. Individuals were excluded if they did not meet these criteria. The research team contacted eligible fathers to schedule interviews. Out of 64 fathers who completed the survey, 43 fathers were contacted and interviewed. Twenty-one fathers did not qualify for the interview study or were contacted and later decided not to participate. Only audio recordings, not video, were collected. Thirty-seven interviews were completed in English and five interviews were completed in Spanish. Interviews were translated, as needed, and transcribed.

The sample includes fathers from across the U.S. Most participants (81%, *n* = 35) were biological fathers. See Table 1 for father sample descriptive information. Most fathers self-identified as White, Black, or Latino, and were born in the U.S. Most fathers reported they worked full time. Fathers reported a range of educational backgrounds, from receiving a degree after college to finishing college to completing some or no college or high school. Most fathers lived with their high school-aged teen. Teens of the interviewed fathers had an average age of 15.53 years (SD = 1.37) and identified as 51% male and 49% female.

### 2.3. Data Collection

Interviews were conducted with 43 fathers in the U.S. from February 2021 to June 2021, when saturation was achieved [36]. Most interviews with fathers lasted approximately one hour, ranging from fifty minutes to one hour and twenty minutes. Interviews were conducted and recorded via Zoom at a time convenient to the father. Four interviewers, including the PI, conducted interviews with fathers. All interviewers had prior experience with qualitative interviewing. Furthermore, the PI trained all interviewers to understand the protocol and sample and addressed interview questions both individually and as a group throughout the interview process. To minimize bias, reliability checks included a team member who did not conduct any project interviews. At the start of the interview, interviewers reminded fathers that the purpose of the study was to understand how families talk about dating, relationships, and sex, and particularly how fathers and teens talk about these topics. They were reminded that these topics can be embarrassing to talk about, and that they may feel a bit uncomfortable, which is normal. Fathers were told they could choose not to answer any questions. They were asked to create pseudonyms to protect their confidentiality; those pseudonyms are used in this article. Interview guides were developed based on prior team parent interview protocols about family talk about sex and research findings on father–teen talk about sex, then modified based on team feedback and fathers’ feedback through pilot interviews. Father interviews included questions about a father’s role in talking with teens about dating, relationships, and sex; the messages he conveyed to his teens; and any barriers to conversations with teens. Fathers were also asked about their attitudes toward a potential intervention to support father–teen talk about dating, relationships, and sex, and feedback about the structure and content of the intervention. Table 2 includes key interview questions.

### 2.4. Data Analysis

Content analysis was used to systematically identify themes in the interview transcripts [37]. Following Schrier’s steps for content analysis [38], overarching themes were identified. These were discussed, revised, and named by the first and second author. We then operationalized each theme, creating a coding scheme by writing up definitions of each theme, containing included and excluded content. We then sought additional examples in other interviews and discussed whether they fit the theme, revising theme definitions throughout the process. The themes were not mutually exclusive, in that one father’s response could generate multiple codes. Only themes that fit with at least 10% of participants’ responses were included. NVivo 12.0 (QSR International: Melbourne, Australia) [39] was used to facilitate coding. We explored how fathers described their role in talking with teens about dating, relationships, and sex; what messages they wanted to share with their teens; how they conveyed these messages; and barriers to their communication with teens. Additionally, we explored fathers’ attitudes toward a potential intervention to support their communication with their teens about dating, relationships, and sex; the structure of the intervention; and what the topics the intervention would address.

### 2.5. Rigor

To support the trustworthiness of study coding, the first and second authors conducted reliability checks for coding, in which reliability equals the number of agreements divided by the total of codes [40]. The researchers coded data in groups of ten participants and discussed inconsistencies after each round of reliability checks. The final intercoder reliability of 94% represented a high-level agreement between the two coders.

### 2.6. Ethical Considerations

This study was approved by the Institutional Review Board of the researcher’s affiliated institution (IRB protocol #201059R, 28 October 2020). All participants gave their informed consent before they participated in the study. Participants were informed about the purpose of the study and the costs and benefits of participation. They were told that participation was voluntary and that they could stop the interview at any time. They were assured that their privacy would be protected. Participants were offered a $50 gift card in appreciation of their study participation.

## 3. Results

### 3.1. Fathers’ Perceptions of Goals, Priorities, and Barriers to Talk

The first four themes relate to participating fathers’ perceptions of their goals and priorities related to talk with teens about sex and the barriers to achieving them. See Table 3 for sample descriptives for each theme and subtheme. The first theme, Father roles in talk with teens, explores how fathers perceived their roles in talking with their teens about sex and relationships. This theme was addressed by all fathers (*n* = 43).

The first subtheme for Father roles in talk with teens was Educator/advisor (53%, *n* = 23) and addressed ways in which fathers perceived their roles as educating, providing support, or giving advice to their teens about dating and relationships. For example, Al explained, “I feel our job, our responsibility, is to teach our kids everything they need to know about the process, about reproduction. Whether that’s a bad relationship, whether that’s just jumping into sex too soon. Whether that’s having a baby when you weren’t prepared to have a baby. Whether that’s, you know, getting a vaccination for HPV, you know, whatever it might be. I feel like a parent’s responsibility, a dad’s responsibility then is to teach your kids all of these things”. Jesus shared, “I think a father, just with anything, is providing guidance and support, um, through any aspect of-of those coming of age years”.

The second subtheme, Parent and teen gender (98%, *n* = 42), addressed how fathers’ gender as well as their teen’s gender reflect how fathers perceived their roles. Specifically, many fathers discussed how they can provide a male perspective to conversations about dating, relationships, or sex and how conversations can be shaped by teens’ gender. AJ talked about the value of his perspective in talking with his daughter: “I think with any, you know, mother, father, household, you know, I think it’s just bringing a different perspective into it. Um, ‘cause you know, obviously I was a 15-year-old boy, granted it was a long time ago, but you know, I think just getting a perspective and point of view, like, hey, this is what, you know, typically boys would like out of a relationship average, you know, good and bad, right?” Dewater discussed how his role was related to his teen’s gender: “I have sons. So I saw that as my responsibility to speak with my boys. Because I think both young people regardless of their-their sexes will feel more comfortable speaking with someone of the same sex when talking about these issues rather than speaking with someone at the opposite sex”.

The third subtheme, Responsiveness to teen (35%, *n* = 15), addresses how fathers explained their roles as being open and honest resources for teens. For example, Billy shared how he tries to be open with his daughter: “I think it’s just a-a father’s role to-to prepare her the best way that-that we can and-and to be there I-with an open mind and-and dialog to-to help them through anything out there that we can… she can come to us and ask us anything and that’s what we’ve always told her”. Tiger also talked about his openness with his son: “He has to be able to come to me, um, without any fear of repercussions. I have to be just an open platform where he can come in and-and-and share his feelings and not feel like he’s being ridiculed or, um, um, you know, pushed in any way. He’s not scared to even ask the weird questions, which-which I love”.

The second theme, Father messages to teens, addresses fathers’ perceptions of the most important messages they wanted to share with their teens. This theme was addressed by 98% of fathers (*n* = 42) and included the key messages fathers wanted teens to know about dating, relationships, and sex.

The first subtheme for Father messages to teens was Healthy relationships (44%, *n* =19). Fathers expressed the importance of teens’ understanding features of healthy relationships and how to support a healthy relationship. James discussed how he talks with his son about healthy relationships: “Well, it’s all about foundation, and as we talked about as well, I always, as a child all the way up to now, I always tell him, treat women that you care for, and/or just women in general, just people in general, the way that you would like to be treated. Don’t treat anyone in a negative fashion or any fashion that-that would be frowned upon. He’s a- he’s a kindhearted kid, and-and I just tell him, ‘Treat everyone with respect.’” Miles said, “I want her to understand what it means, um, to have a healthy relationship, the different components that-that-that would entail. You know, not just, you know, learning how to discuss about, um, any arguments or problems that they might have, but also like what a healthy person looks like. And, you know, like, does that person deserve to be verbally abused? Does she deserve to be verbally abused, right? And understanding why nobody deserves any of that”.

The second subtheme, Preventing pregnancy and STIs (37%, *n* = 16)*,* addresses the importance fathers attributed to talk with their teens about preventing pregnancy and STIs. For example, Maurice said, “I want her to know about all the sexually transmitted diseases. I want her to know that protection it, um, it helps, you know. I want her to practice safe sex”. Julio mentioned how he talks with his son about preventing pregnancy: “That he is always well protected, um, whether or not if he has a partner, that-that they plan everything. Not just when they do things to do them and then the consequences come”.

The third subtheme, *Boundaries and consent* (21%, *n* = 9), addresses the value fathers placed on helping their teens to understand how to be comfortable with relationship boundaries and not feel pressured to engage in sex. George describes his messages to his son about boundaries: “Just how important it is to be able to, um, communicate and be friends with somebody that you’re in a relationship, whether it’s sexual or not, and to respect their boundaries and also to be very comfortable with expressing what his boundaries are”. Similarly, Ted described his conversations with his daughter about feeling pressured: “She shouldn’t feel pressured to do anything that she doesn’t wanna do. Um, you know, and any bit of awkwardness,—You know, say no, and it’s okay to do that and- uh, and you know, if she ever feels, uh, in-in like an awkward situation that she should, tell us about it and just talk about it, but, uh, just to kind of, you know, just to respect herself”.

The third theme, Father approaches to talk with teens, addresses how fathers approach talk with teens about sex. This theme was described by 88% (*n* = 38) of fathers.

The first subtheme of Father approaches to talk with teens is Direct communication (58%, *n* = 25). These fathers reported using clear and straightforward communication with their teens about sex and relationships. Derek said, “With her, I’ve been direct. I’ve spoken plainly”. Similarly, Vic said he uses “consistent conversation” when talks with his daughter.

The second subtheme is Indirect communication (19%, *n* = 8), which involves fathers talking in a vague way about sexual topics or using a family or friend experience, movie, or TV show to start a conversation. For example, James said how he uses similar interests to start the conversation: “Well, we both have a lot of the same interests. So, while we’re- like, we both love video games, or we both love music. So, if we-we’re just sitting around listening to some music or playing the games, if something comes up, you know, during the game, someone says something online that’s, you know, ugly or mean, you know, it may segue us into a conversation”. Pedro T explains how he talks more generally with his son: “I always tell him about- about life in general, but not-not exactly like, ‘Oh yeah, you know what, um, I want you to be careful when you’re dating or when you’re having sex or you have, uh, you know, sexual relationships,’ or whatever. Uh, I just most likely, uh, talk to him in general. Not- not- not just, you know, relationship and sex”.

The third subtheme, Lead by example (14%, *n* = 6), addresses how some fathers describe conveying their messages to teens about sex and relationships through actions, such as modeling healthy behaviors. Tiger described, “I use myself as an example and the way that I, um, speak and the way that I treat, uh, their mother, um, I always try and set an example to them is that this is how you treat a woman”. Similarly, Jeff mentioned, “I’ve done a good job now modeling”.

The final theme related to fathers’ perceptions of their goals and priorities, Barriers to talk, addresses how fathers describe what interferes with their communication with their teens about sex. This theme was addressed by 98% (*n* = 42) of fathers.

The first subtheme for Barriers to talk is Father/teen discomfort or avoidance (47%, *n* = 20). This subtheme addresses when fathers described either themselves or their teens as feeling uncomfortable or trying to stay away from conversations about sex or relationships. Rob talked about how his own discomfort interfered with talking with his son about sex: “Maybe I overthink and just avoid the opportunities because I’m worried that it’s not gonna be received well, or I’m not- I’m-I’m not gonna be succinct about it”. Edward described how his daughter’s avoidance impacts their conversations: “I wouldn’t say they don’t go so well, but she definitely tries to avoid the conversation of sex. It’s probably uncomfortable. Yeah. Awkward”.

The second Barriers to talk subtheme is Teens’ lack of readiness or interest (16%, *n* = 7), which addresses fathers’ perceptions that teens were not developmentally prepared for talk about sex were not engaged with or invested in dating or relationships. For example, Derek T said, “I keep saying, ‘Oh, she’s not dating. It doesn’t necessarily need to be spoken about yet.’” AJ mentioned why he doesn’t think his daughter is ready: “I could be obviously co-completely off base here but I just, you know, kinda, what I mentioned upfront, I just, I don’t think it’s-it is, it’s, I mean, I-I have a feeling it’s probably something she thinks about but it’s just not like a priority of hers right now so it’s just you know having discussion about nothing at this point”.

The third Barriers to talk subtheme is Hard to begin conversations (19%, *n* = 8), in which fathers described their difficulties in starting a conversation with their teens about sex or relationships, often because they felt unprepared or did not know what to say. David described how conversations are hard for him: “I don’t know why, but generally, I stay away. It’s kind of the topics. I don’t know what to talk, what should I say?” Roberto talked about what gets in the way for him: “I don’t know, I-I-I’m-ashamed, I would like to be eh- better prepared…from my time to the time of my girl, right? They are totally different times. You have to be prepared, I think, to be able, this, to speak, right? So openly”.

The fourth Barriers to talk subtheme is No barriers (21%, *n* = 9), in which some fathers reported that they did not experience barriers to talk. For example, Vic said, “I-I don’t avoid any conversations and, uh, t-those conversations have always kinda gone well. W-whenever we have those conversations they go well”. Similarly, Cesar said, “So far all conversation so far do work”.

### 3.2. Fathers’ Intervention-Based Feedback

The last three themes address fathers’ perspectives on what they would look for in an intervention to support their talk with their teens about sex and relationships. The first intervention-based theme, Attitudes toward intervention, includes participants’ opinions about a potential program to support fathers’ talk with their teens about sex and includes both fathers’ own perceptions and those of others. This theme was addressed by almost all participants (95%, *n* = 41).

The first subtheme for Attitudes toward intervention is Positive attitudes, which includes fathers’ encouraging comments about a potential intervention to support fathers’ talk with their teens about sex and relationships. Ninety-one percent (*n* = 39) of participants expressed positive attitudes toward an intervention. Maurice shared why he thought a program would be helpful: “I think that (a program) will be a great help because I’m strong and I’m blunt and I’m straightforward and all that. But some of those conversations, I just can’t jump in like that. Like, uh, I don’t want to talk about that. Like, I don’t want to talk about sex-sex, like, you know, sex-sex. Like, I don’t want to talk about that with my daughter, my stepdaughter. Shoot, even my oldest son, I don’t want to have those conversations, but I know they are necessary”. Leo stated, “It would help me answer some of the questions I had on how to talk to him (his son), how to bring subjects up. I’m a single father and I have nobody to talk to. I don’t have family to talk to. And it’s not something you bring up to many friends to talk to about…just having the birds and the bees talk, you know, it’s, some, for some parents it’s hard”.

The second subtheme for Attitudes toward intervention was Negative attitudes, which focused on fathers’ discouraging comments about a potential intervention. Twenty-six percent (*n* = 11) of fathers expressed negative attitudes about an intervention program, often describing a lack of need for a program or potential concerns that other fathers might have due to cultural or religious values. Michael shared why a program would not be relevant for him: “I mean, me personally, I feel like I have a big support system. I have, you know, my parents, my brothers, my sister, friends, and cousins and so I always have someone. But someone who maybe doesn’t, they would probably benefit more than I would”. Other fathers raised concerns that might prevent some fathers from participating. Dewater said, “Some parents would not be accepting of programs, uh, that taught things about, you know, sexual orientation and those kinds of issues”.

The theme Feedback on intervention structure addresses fathers’ expressed preferences for how an intervention would be set up. This theme included 84% of fathers (*n* = 36) and addresses the logistical set-up of a program, issues related to program leadership, and what kinds of tools and resources fathers think would be useful to share with program participants.

The first subtheme for Feedback on intervention structure is Program delivery, which addresses the importance of conversations with other fathers, in order to learn from each other and share experiences. Most fathers (74%, *n* = 32) discussed the modality of the program. For example, Felix described a model for peer learning: “NFL players come into a training camp, you have rookies, you have, you know, mid-level guys, and then you have the old grizzly veterans, and, um, and they all share information with each other and learn from one another. I think that it’s important for men to be able to learn from other men and to share their experiences so that they can improve each other”. Al also talked about the importance of sharing with other fathers: “Having that ability to talk to a dad as a friend, nonjudgmental, gonna listen to me and just be able to say, ‘Dude, I’m running into this. I don’t know how to talk to my son or daughter about sex.’”

The second Feedback on intervention structure subtheme is Program leadership, which often focused on the importance of having a leader with similar background or experience to the participants. This subtheme was addressed by 35% of fathers (*n* = 15). For example, Jerry explained, “I’m tatted up, gauges, dreads all this type of stuff you know. And a lot of young- even- if it is that you’re in black, white young boys whatever, they will feel more comfortable speaking to somebody like me versus somebody that’s in a suit”. Similarly, Marcell stated, “Not corporate (leadership). I understand these people are parents as well, but not corporate suit and tie, people necessarily, unless that’s the people you’re trying to reach, but if you’re trying to reach like the community and regular people like me or whatever, you-you, it would be dope if you seeing regular people, you know?”

The third Feedback on intervention structure subtheme is Program resources, which includes requests for access to information and opportunities to ask questions. This subtheme was addressed by 35% of fathers (*n* = 15). Some participants suggested that simple and easily accessible information was key to its effectiveness. Roger commented, “some scenario-based curriculum, um, maybe some printable materials, especially when it comes down to, um, facts around, um, sexually transmitted diseases and so on and so forth…if we have like a search engine or something and then I have a question then I can- I can go onto that search engine and-and get results”. Al talked about the importance of clear and simple direction: “Make it as user-friendly as-as-as possible. Um, you know, just something very easy, quick hit, um, you know, don’t make it complicated. You want to give me a manual, like five steps and this is what I do. And that’s how I talk to my kids… I think having the ability to-to be anonymous, you know, I think it might be helpful, ‘cause I mean, it’s-it’s hard to ask a question, you know, it’s hard to ask for help on some of these topics, you know, um, maybe realizing that you don’t know-know all this”.

The final theme was Feedback on intervention content and process, which describes fathers’ priorities for what topics and skills should be addressed in an intervention program. This theme included 95% of fathers (*n* = 41).

The Content subtheme addressed why fathers perceived certain topics as important to discuss in an intervention program. This subtheme was addressed by 93% of fathers (*n* = 40). Many fathers discussed the importance of talking about topics which have potential to derail teens’ lives by threatening their health, safety, or education. Felix shared, “I just see so often how choosing a wrong person to have a relationship with and then subsequently have children with, could completely change the trajectory of someone’s life. And, um, and so those coming up with uh, solid ways to make decisions around, um, relationships and, uh, and know who you’re having sex with and-and having children with, those are so critical to making sure that, uh, you’re able to accomplish things that you want to do with your life”. Derek focused on the consequences of teen pregnancy or getting an STI: “These kids have their whole life ahead of them and the last thing they wanna do is have something like-like a pregnancy- like a pregnancy, deter them from getting their goal- getting their goal…you’re so young and you still have the rest of your high school, your college, and the rest of your life to figure out. Don’t ruin it by getting the lifelong disease or having to drop out of school just to take care of the baby or anything of those sorts”.

The Process subtheme focused on skills fathers identified as important to learn from an intervention program. This subtheme was addressed by 35% of fathers (*n* = 15). Many fathers talked about the need to learn how to talk with teens about sex and relationships, including when and how to start a conversation with a teen. Pedro explained, “I still don’t know, uh, what is right, what is enough, uh, how much, uh, what level or to what extent, uh, the talk is okay, right? I just probably would (want to) know from someone, this is probably a proper way of talking and to what extent you can talk to your kids, you know. It would be, yeah, it would be good to know, rather than being, you know, entirely clueless”. Ted talked about the need for guidance on how to begin a conversation, “something that gives you discussion topics or, you know, or different tactics to generate-to initiate these discussions…More tricks, more tools, more things to help to get the conversation going. So, just, you know, just kinda keep that in mind like things to ease awkwardness”.

## 4. Discussion

Following the PRECEDE model, this paper provides input from fathers with teen children on their goals and priorities related to talking with their teens about sex and relationships as well as barriers to having these conversations. In order to guide next steps in intervention development, this paper also includes fathers’ feedback on what they would look for in a program to support health-promoting talk with their teens about sex and relationships. This is one of few studies which provides a needs assessment for fathers’ talk with their teens about sex and relationships as well as an exploration of what fathers would look for in an intervention.

Fathers described their roles in talking with their teens about sex and relationships as both educators and open and responsive listeners. These roles fit with what adolescents and young adults look for in talk with parents about sex [41,42,43]. These findings fit with prior studies which found that fathers view talk with their teens about sex as an important role for fathers [16,44]. Participating fathers also discussed the importance of both parent and teen gender in shaping their roles in talking with teens about sex. Many fathers shared a belief that their perspectives and experiences as males could bring useful insight about sex and relationships to male and female teens. However, they also described ways their teens seemed more comfortable talking with same-gender parents about sexual issues, suggesting more paternal responsibilities toward sons than daughters. The focus on same-gender communication fits with research showing lower levels of father–daughter than father–son talk about sex [4,15], and suggests that fathers may feel more responsible for sons than daughters [14]. However, fathers’ identification of a unique role for sharing male perspectives identifies potential for support for their teens regardless of teen gender.

The messages fathers described as important to share with their teens focused on key issues related to teens’ health, including healthy relationships, preventing pregnancy and STIs, and boundaries and consent. The first two topics have been found in prior research [11,12], but the focus on consent is less documented in fathers’ or mothers’ talk with their teens, as parents may see it as taboo to discuss [45]. Fathers’ recognition of the importance of this issue is meaningful, since most school-based sex education programs do not address it [46]. The topics fathers identified as important to discuss are consistent with fathers’ feedback on the need to address topics in an intervention program that could threaten their teens’ health and future, such as unhealthy relationships or teen pregnancy. However, one topic which was virtually absent from fathers’ feedback on content for an intervention was sexual orientation and identity. Despite growing acceptance of gay marriage in the U.S. in recent decades [47], parents often assume their children are heterosexual [48] and are reluctant to discuss sexual orientation with their children [11].

Fathers’ descriptions of how they approach talk with teens about sexual issues showed both similarities and differences from mothers’ talk with teens. Direct talk, the most commonly described type of communication in this study, fits with prior findings on how parents (largely mothers) tend to communicate with teens about sex [49], and on how communication about sex is typically measured [5]. The percentage of fathers in this study who describe direct talk is higher than prior research [4,15,30], which likely reflects disproportionately high engagement in family communication among fathers who volunteered for this study. Fathers’ descriptions of indirect talk and role modeling as ways to communicate with teens suggest that some fathers may engage differently with teens than mothers around sexual issues. A quantitative study found that more than half of teens reported indirect talk with their fathers about sex [30], an approach which may provide a way to manage discomfort in talking about sexual issues [50]. Interventions geared toward fathers may benefit from addressing multiple ways that fathers approach communication with their teens.

Fathers identified barriers to talk with teens about sex and relationships, including parent or teen discomfort, teens’ lack of readiness for these conversations, and challenges of how to start a conversation with a teen about sex. These barriers are consistent with prior research on fathers [16,17]. Perceptions of teens’ lack of readiness to talk about sex has also been identified for mothers, and often focuses on girls, which may indicate parents’ reluctance to see their daughters as sexual beings [51]. Furthermore, delaying talk about sex due to teens’ perceived lack of readiness can keep teens from accessing needed information, as many parents under-estimate their teens’ involvement in sexual activity [52,53]. Finally, fathers’ descriptions of their struggles with how to start conversations with their teens about sex matches their feedback on the need for intervention programs to address *how* to talk about sex, not just provide information about *what* to discuss with teens.

Fathers’ positive attitudes toward a potential intervention to support father–teen talk about sex and relationships is striking, and largely reflected fathers’ descriptions of how hard it is to talk with teens about these topics and their desire for support in this area. They also relate to the lack of sex education programs that tailor their approaches to fathers. While few fathers described negative perceptions of potential intervention programs, those who did often discussed cultural or religious concerns. Interestingly, studies suggest that many religious parents support sex education programs [54,55], although religious values may support a parental focus on talk with teens about abstinence [44].

Fathers who shared feedback on what structures they wanted to see in an intervention program showed some consistent patterns. Many fathers discussed the importance of talking with other fathers to share experiences and learn from each other, with easy access to information and opportunities to ask questions. This feedback speaks to the value fathers attribute to an interactive group setting in which fathers with shared experiences can learn together. Several fathers expressed a desire for program leadership from people with similar backgrounds to fathers in the group, such as a program that includes barbers as facilitators for an HIV prevention program for African American men [56].

As with fathers’ goals for their own conversations with their teens, fathers identified important program content areas as those they perceived as key to their teens’ long-term health, safety, and educational outcomes. Fathers talked about the importance of discussing topics that are essential to protecting their teens from experiences such as poor relationships, teen pregnancy, and STIs. These content areas are consistent parent–teen communication about sex and relationships [49]. An area less addressed in existing parent programs is a focus on *how* to talk with teens about sex and relationships, a need identified in a feasibility study of a reproductive health intervention for Latino fathers [8]. Findings from a study of sexual communication between African American fathers and their sons also identified fathers’ interest in learning how talk with their sons about these issues [18]. Many fathers requested guidance on how, when, and how often to talk with teens about sexual issues. This feedback suggests the need for intervention programs to provide tools to support both the content and process of talk with teens.

Key limitations of this study include the sample of highly engaged fathers, who likely talk more with their teens about sex and relationships than a more representative sample. Studies that include fathers who are less engaged in family talk about sex would be useful, but may be difficult to conduct due to the challenges of recruiting fathers to social science research [57]. Social desirability may have led fathers to present themselves in ways they perceive as more favorable to the interviewer. Although this study’s sample is racially diverse, additional research is needed to discern differences in communication based on racial/ethnic background. In addition, given fathers’ focus on father and teen gender in shaping their roles in family talk about sex and prior research identifying the impact of gender in family talk about sex [5,49], more gender-based studies are needed, particularly to explore similarities and differences in how fathers talk with male, female, and transgender teens, and their implications for intervention.

## 5. Conclusions

This study provides a unique lens into fathers’ views of their roles in family talk about sex, the barriers which inhibit these conversations, and what fathers look for in a potential intervention to support their communication with teens about sex and relationships. This needs assessment suggests that many fathers see talk with teens about sex as part of their role as parents. While fathers’ perceived roles, key messages to teens, and barriers have much in common with mothers, fathers also view their roles as highly gender-based and a unique contribution to family communication. Furthermore, some fathers described indirect talk and role modeling approaches to communication, a difference from mothers’ typical focus on direct talk [49]. These findings suggest the need for interventions which provide overlapping yet distinct elements, compared to existing programs to promote parent–teen communication about sex, which are largely based on mothers [3]. What fathers look for in the content of an intervention is similar to existing programs [58,59]; their feedback on program structure and process paints a picture of an intervention that is more peer-based and interactive than many existing sex education programs, and addresses the *how* in addition to the *what* for talk about sex and relationships.

Overall, the findings from this study show the need for programs to support fathers’ talk with their teens about sex and relationships. The potential of father–teen talk about sex to protect teens’ sexual health, along with low levels of fathers’ talk with teens about sex and relationships and a lack of existing programs tailored to support father–teen communication about sex, show a need to develop or revise intervention programs with a focus on fathers. Fathers’ positive attitudes toward a potential intervention program and their feedback about what characteristics they would look for in a program provide guidelines for characteristics of a program that could feel useful and engaging to fathers.

## Figures and Tables

**Table 1:** Father sample descriptives.

Father Sample Descriptives	Number	Percent
Region		
West	17	40
Midwest	14	33
South	11	26
Northeast	1	2
Father Relation		
Biological	35	81
Step	5	12
Adoptive	3	7
Race/Ethnicity		
White	16	37
Black	14	33
Latino	10	23
Asian	2	5
Middle Eastern	1	2
Born in the U.S.	32	74
Work status		
Full time	35	81
Part time	3	7
Not working	4	10
Prefer not to answer	1	2
Education		
Less than high school	4	9
Completed high school	9	21
Some college	8	19
Completed college	9	21
Degree after college	12	28
Prefer not to answer	1	2
Lives with teen	34	79

**Table 2:** Key interview questions.

Key Interview Questions
Fathers’ perceptions of goals, priorities, and barriers to talk
What are the most important things you want (teen’s name) to know about dating, relationships, or sex? How have you been able to pass on those messages?
In general, what do you think a father’s role should be when it comes to talking about dating, relationships, and sex? How does that fit with how you are with (teen’s name)?
What kinds of conversations with (teen’s name) about dating, relationships, or sex don’t go so well? Are there conversations you avoid? Why is that?
Fathers’ intervention-based feedback
What would you think about a program that would support your talk with (teen’s name) about dating, relationships, and sex?
Would you be open to a program like that? Why you would be open/not open to this program?
What topics do you think are most important to include in a program for fathers? Why do you think it’s important to include these topics?
What should a program like that look like? Why do you think that?

**Table 3:** Father perception and intervention feedback themes and subthemes.

Father Perception and Intervention Feedback	All Fathers (*n* = 43)	
	Number	Percent
**Father roles in talk with teens**	43	100%
Educator/advisor	23	53%
Parent and teen gender	42	98%
Responsiveness to teen	15	35%
**Father messages to teens**	42	98%
Healthy relationships	19	44%
Prevent pregnancy and STIs	16	37%
Boundaries and consent	9	21%
**Father approaches to talk with teens**	38	88%
Direct communication	25	58%
Indirect communication	8	19%
Lead by example	6	14%
**Barriers to talk**	42	98%
Father/teen discomfort or avoidance	20	47%
Teens’ lack of readiness or interest	7	16%
Hard to begin conversations	8	19%
No barriers	9	21%
**Attitudes toward intervention**	41	95%
Positive	39	91%
Negative	11	26%
**Feedback on intervention structure**	36	84%
Program delivery	32	74%
Program leadership	15	35%
Program resources	15	35%
**Feedback on intervention content and process**	41	95%
Content	40	93%
Process	15	35%

## Data Availability

The data presented in this study are available on request from the corresponding author. The data are not publicly available due to concerns about privacy of participants’ responses.
